# The Value of Diffuse Splenic and Hepatic ^18^F-FDG Uptake on PET/CT in Diagnosing Patients with Anemia

**DOI:** 10.1155/2022/3804673

**Published:** 2022-02-23

**Authors:** Li Deng, Lin Liu, HaoYuan Ding, ShuMao Zhang, Wei Wang, Wei Zhang, Yi Li

**Affiliations:** ^1^Department of Dermatology, The Affiliated Hospital, Southwest Medical University, Luzhou 646000, Sichuan, China; ^2^Nuclear Medicine and Molecular Imaging Key Laboratory of Sichuan Province, No. 25 Taiping St, Luzhou 646000, Sichuan, China; ^3^Department of Nuclear Medicine, The Affiliated Hospital, Southwest Medical University, Luzhou 646000, Sichuan, China; ^4^Department of Nuclear Medicine, Sichuan Provincial Peoples Hospital, University of Electronic Science and Technology of China, Chengdu 610072, Sichuan, China

## Abstract

**Objective:**

Anemia is a disease with a negative impact on the progression and prognosis of tumor diseases and usually diagnosed by blood tests. Imaging examination has been used as an alternative method to diagnose anemia in addition to blood tests for patients who cannot tolerate blood draw (such as those with severe coagulopathy). The purpose of this study was to investigate the role of diffuse splenic and hepatic ^18^F-FDG uptake on PET/CT in anemia, by analyzing the correlation between the hemoglobin level and diffuse splenic and hepatic as well as marrow ^18^F-FDG uptakes in patients who underwent PET/CT.

**Materials and Methods:**

Forty four patients who underwent a peripheral blood examination within 2 days of a ^18^F-FDG-PET/CT in our hospital from March 2020 to March 2021 were included. The standardized uptake value (SUV) of the spleen, liver, and marrow were measured, including the maximum value (SUVmax) and the mean value (SUVmean), and the CT value (CTV) of the left ventricular (LV) cavity was measured, including the maximum value (CTVmax) and the mean value (CTVmean). The relation between these measurements and the blood hemoglobin level were analyzed.

**Results:**

Our analysis revealed that the hemoglobin level was negatively correlated with the SUVmax of the spleen (*P* ≤ 0.01, *R* = −0.385), SUVmean of the spleen (*P* ≤ 0.01, *R* = −0.395), SUVmax of the liver (*P* ≤ 0.05, *R* = −0.365), and SUVmean of the liver (*P* ≤ 0.05, *R* = −0.315). The hemoglobin level was positively correlated with CTVmax of the LV cavity (*P* ≤ 0.05, *R* = 0.33) and CTVmean of the LV cavity (*P* ≤ 0.05, *R* = 0.382), while no statistically significant correlation between the hemoglobin level and the SUV of marrow was observed (*P* > 0.05).

**Conclusion:**

Our study revealed a negative correlation between the hemoglobin level and spleen SUV as well as liver SUV, and a positive correlation between the hemoglobin level and CTV of the LV cavity. These findings may provide potential indictors for the imaging diagnosis of anemia, which has important clinical significance in certain clinical scenarios including the evaluation of anemia status in patients who cannot tolerate blood draws and retrospective clinical studies based on patient imaging data.

## 1. Introduction

Anemia is a disease with an incidence of about 24.8% and increased risk with older population. It is one of the main factors leading to the global burden of disease [1,2]. Anemia is defined as a condition in which the body has a lower number of erythrocytes or hemoglobin or hematocrit in the circulating blood than normal. The World Health Organization (WHO) defines anemia as a hemoglobin level less than 13 g/dL in adult men and less than 12 g/dL in nonpregnant adult women [3]. It has been subdivided into mild (9 g/dl—normal), moderate (6–9 g/dl), and severe (<6 g/dl).

Although anemia can be diagnosed through blood tests in most cases, the diagnosis of anemia by blood tests has certain limitations, especially for patients who cannot tolerate blood draw (such as severe coagulopathy), follow-up of outpatients without obvious clinical manifestation of anemia, and large-scale retrospective study in which blood draw is missing or unavailable. Imaging diagnosis can be used as an alternative diagnostic method for anemia. However, despite previous studies have suggested that the CT attenuation of the LV cavity showed a strong correlation with the peripheral blood hemoglobin and the distinct morphological feature of ventricular wall in patients with severe anemia [4–6], the assessment can be unreliable when patients have diseases that affect cardiac density, such as vasculitis, glycogen accumulation, or hepatic hemochromatosis. Thus, the imaging diagnosis of anemia remains to be improved with new and reliable indictors.

^18^F-FDG-PET/CT plays an important role in the diagnosis and staging of tumors as well as the evaluation of efficacy and prognosis. It also plays an important role in other non-neoplastic diseases. There are some research studies that indicated patients with anemia may have a diffuse splenic and hepatic ^18^F-FDG uptake on PET/CT [7–9], but the relationship between the SUV of the spleen or liver and anemia is still undefined. In this study, we investigated the value of ^18^F-FDG-PET/CT in the diagnosis of anemia.

## 2. Materials and Methods

### 2.1. Patients

44 patients who underwent ^18^F-FDG-PET/CT from March 2020 to March 2021 and had peripheral blood examination within 2 days were included in this study. The study was performed retrospectively. With the approval of the ethics committee of our hospital, the patients were notified in written format, and their medical information was anonymized for research.

In this study, we defined anemia as hemoglobin level <130 g/l in males and <120 g/l in females [3]. Hemoglobin concentration at (90—normal) g/l was defined as mild anemia (14 patients); (60–90) g/l was defined as moderate anemia (23 patients); and less than 60 g/l was defined as severe anemia (4 patients). In addition, our study included 3 patients with normal hemoglobin level.

The exclusion criteria are as follows: (1) patients with localized lesions in the spleen and liver; (2) patients without peripheral blood examination within 2 days after PET/CT exam; (3) patients who had undergone abdominal enhanced CT or enhanced MRI within 2 days after PET/CT exam; and (4) patients with hematological diseases, such as lymphoma and leukemia.

### 2.2. Diagnosable Image Quality

PET/CT (Gemini TF, Philips, USA) with 16-detector row was used. ^18^F-FDG is produced by the cyclotron of our department and used after quality control. Before the ^18^F-FDG injection, patients were instructed to fast for at least 6 hours, and the blood glucose level was confirmed to be less than 10 mmol/L. Each patient was injected with ^18^F-FDG at a dose of 0.15 mCi/kg. After the injection of ^18^F-FDG, patients were instructed to rest for 55–75 minutes before the scan. Firstly, low-dose CT with FOV600 mm, voltage 120 kV, current 100 mAs, layer thickness 5 mm, layer spacing 5 mm, and pitch 0.813 was used to scan from the top of the skull to the upper femur. Subsequent PET/CT (FOV576 mm) was used, by step-by-step acquisition, 70S/beds, including 9–10 beds. OSEM (ordered subset maximum expected value method) reconstruction was performed after the acquisition, of which the subsets were 33, iterations were 3, and the matrix was 144 × 144. Finally, the Medex fusion software was used for image analysis.

### 2.3. Image Analysis

The PET images of all patients were interpreted by two experienced nuclear medicine physicians who were blinded to the outcomes of peripheral blood examination. Regions of interest (ROIs) confined to the spleen, liver and bone marrow were drawn by referring the CT and PET/CT fusion images for positioning. The SUVmax and SUVmean were measured and defined as spleen SUVmax, spleen SUVmean, liver SUVmax, liver SUVmean, marrow SUVmax, and marrow SUVmean. The liver SUV was measured in ROIs placed on the right lobe of the liver. The ROI was placed in the middle part of the organs to prevent respiratory motion-induced artifacts and uptake by adjacent organs. When measuring the SUV of the spleen, we referred to the CT image to select the local clearest and largest PET transverse image. When measuring the CT value of the LV cavity, we selected the CT cross-sectional image with the most uniform and largest slice of the LV cavity and expanded the radius as far as possible while avoiding the large blood vessels and ventricular walls.

### 2.4. Statistical Analysis

The statistical analyses were carried out using the SPSS version 23 for Windows software (SPSS Inc, Chicago, IL, USA). Spearman correlation coefficients (*r*) were calculated for the SUV of spleen, liver, and marrow and for the CTV of the LV cavity with regard to the hemoglobin level. Statistical significance was defined as a *P* value of ≤0.05.

## 3. Results

The cohort consisted of 19 males and 25 females with a mean age of 58 years (19–92 years), of which 25 were ≥60 years old, and 19 were younger than 60 years. The peripheral blood hemoglobin level varied from 54 to 169 g/l (mean 85 g/l). 7 patients suffered from colon cancer, 4 patients suffered from esophageal cancer, 8 patients suffered from cervical carcinoma, 14 patients suffered from lung cancer, 5 patients suffered from gastric cancer, 1 patient suffered from pneumoconiosis, and 1 patient suffered from intracranial tumor, while no patient was diagnosed with hematological diseases, such as lymphoma and leukemia. The patients' characteristics are shown in Table 1.

**Figure 1:**
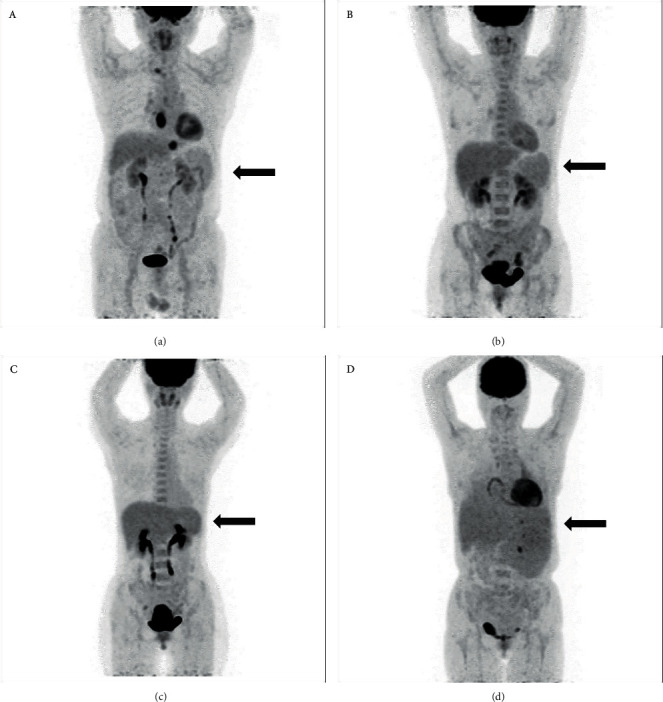
Four cases with different hemoglobin levels, from 56 g/L(A) to 136 g/L(D). The hemoglobin level decreased, as the splenic ^18^F-FDG uptake increased. The patient is male and 71 years old whose hemoglobin level is 136 g/(L). The spleen SUVmax and spleen SUVmean of this patient were 1.436 and 1.183, respectively (a). The patient is female and 42 years old whose hemoglobin level is 106 g/(L). The spleen SUVmax and spleen SUVmean were 1.993 and 1.656, respectively (b). The patient is female and 42 years old whose hemoglobin level is 77 g/(L). The spleen SUVmax and spleen SUVmean were 1.954 and 1.635, respectively (c). The patient is female and 52 years old whose hemoglobin level is 56 g/(L). The spleen SUVmax and spleen SUVmean were 2.16 and 1.787, respectively (d).

**Figure 2:**
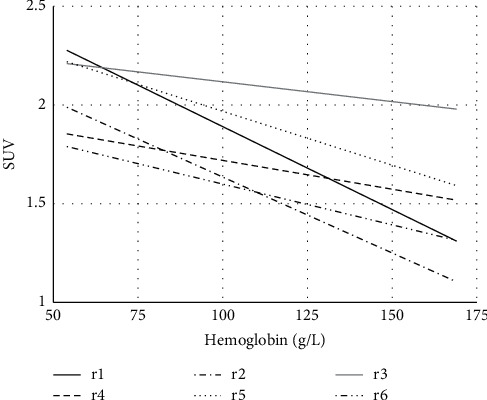
Correlations between the hemoglobin level and spleen SUV, liver SUV, and marrow SUV in patients (spearman correlation coefficient, *r*1 = spleen SUVmax, *r*2 = spleen SUVmean, *r*3 = marrow SUVmax, *r*4 = marrow SUVmean, *r*5 = liver SUVmax, and *r*6 = liver SUVmean).

**Figure 3:**
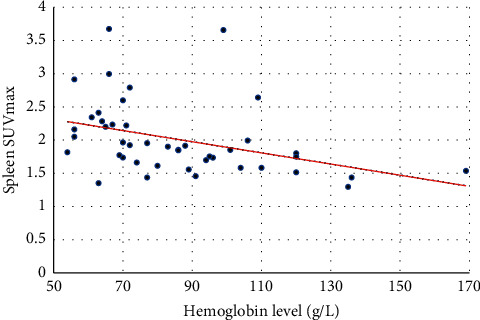
Correlation between the hemoglobin level and spleen SUVmax.

**Figure 4:**
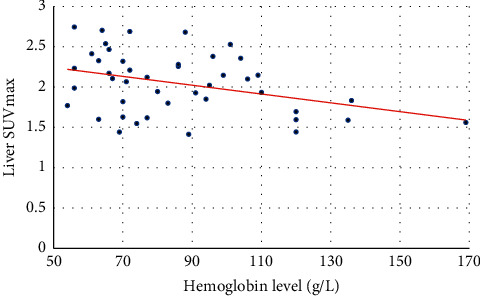
Correlation between the hemoglobin level and liver SUVmax.

**Figure 5:**
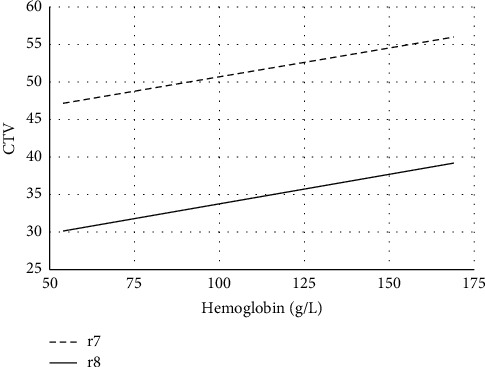
Correlations between the hemoglobin level and CTV of the LV cavity (spearman correlation coefficient, *r*7 = CTVmax of the LV cavity and *r*8 = CTVmean of the LV cavity).

Table 2 shows the ^18^F-FDG uptake of the spleen, liver, and marrow, the CTV of the LV cavity, the hemoglobin level of 44 cases, and the correlations between the hemoglobin level and spleen SUV, liver SUV, marrow SUV, and CTV of the LV cavity of the 44 cases.  Figure 1 shows the FDG uptake of the spleen in 4 patients with different levels of hemoglobin. With the decrease of hemoglobin, the diffuse splenic FDG uptake on PET/CT increased gradually.  Figure 2 is a trend-lines chart showing the correlation between the spleen SUV, liver SUV, marrow SUV, and hemoglobin level.  Figure 3 is a scatter plot chart showing the correlation between the hemoglobin level and spleen SUVmax.  Figure 4 is a scatter plot chart showing the correlation between the hemoglobin level and liver SUVmax.  Figure 5 is a trend-lines chart showing the correlation between the CTV of the LV cavity and hemoglobin level. The correlation between the hemoglobin level and spleen SUV, including spleen SUVmax and spleen SUVmean, was statistically significant (*P* ≤ 0.01). The correlations between the hemoglobin level and liver SUV and CTV of the LV cavity were statistically significant (*P* ≤ 0.05). The correlation between the hemoglobin level and marrow SUV was not statistically significant.

## 4. Discussion

Anemia may severely impair the health condition of patients and have a negative impact on the progression and prognosis of the diseases, especially in the tumor patients [1, 2]. Our study investigated the correlation between diffuse splenic and hepatic ^18^F-FDG uptake and hemoglobin level in patients who underwent PET/CT examination and observed a negative correlation between the hemoglobin level and spleen SUV and liver SUV. These results emphasized the value of diffuse splenic and hepatic ^18^F-FDG uptake in PET/CT in diagnosing patients with anemia, especially for outpatients without routine blood tests and no obvious clinical manifestation of anemia. Our study may have potential clinical significance for the early diagnosis, treatment of anemia, and the prognosis of tumor disease.

Anemia is usually diagnosed by blood tests. However, imaging evaluation methods have important application in certain clinical scenarios, such as patients who cannot tolerate blood draw (such as severe coagulopathy) or underwent emergency chest CT examination or large-scale retrospective study. The traditional imaging method for diagnosing anemia is measuring the CTV of the cardiac cavity. Patients with severe anemia have been found with a reduced cardiac cavity density on the CT scan and distinct morphological feature of the ventricles and ventricular wall [10]. Our study demonstrated a positive correlation between the hemoglobin level and CTV of the LV cavity. In consistent with the results of the previous reports, severe anemia can be evaluated by measuring the CTV of the cardiac cavity [6, 11–13]. However, the diagnosis of anemia by the CTV of the cardiac cavity has certain limitations. The CTV may be inconsistent on different types of CT scanners, due to different scanning parameters [12–15]. Jung et al. [16] also found that patients with atherosclerosis and mural calcification may influence CT attenuation when vascular calcification occurred at the same height of the CT scan. Moreover, the results can also be unreliable when patients have diseases that affect cardiac density, such as vasculitis, glycogen accumulation, or hepatic hemochromatosis. The ^18^F-FDG-PET/CT has been wildly used clinically because of its advantage in displaying the metabolic and functional abnormalities that occurs before the anatomical changes. Therefore, ^18^F-FDG-PET/CT is mainly applied to early detection and diagnosis of tumors and cardiovascular and cerebrovascular diseases [17–20].

At present, the relationships between the uptake of ^18^F-FDG in the spleen, liver, and marrow and anemia remains undefined. However, some studies found that the increased uptake of ^18^F-FDG in the spleen is related to the decreased hemoglobin level, which may be caused by extramedullary hematopoiesis [21–24]. It is well known that the liver is an extramedullary hematopoietic organ, so erythrocyte production in the spleen and liver may increase because of extramedullary hematopoiesis when patients suffered from anemia, which may lead to diffuse splenic and hepatic ^18^F-FDG uptake on PET/CT. The research of Nam et al. [25] showed that there was a negative correlation between diffuse splenic ^18^F-FDG uptake and several hematological indicators, especially hemoglobin concentrations. The results of Pak et al. [26] also showed that ^18^F-FDG uptake in the spleen was negatively correlated with hemoglobin concentrations. The previous studies revealed a negative correlation between the hemoglobin level and spleen SUV/liver SUV ratio because the liver SUV is often widely used as a reference factor due to the homogeneity of the ^18^F-FDG uptake in the liver [26–30]. The marrow is an important hematopoietic organ and may have increased ^18^F-FDG uptake in patients with anemia because of medullary hematopoiesis. Adams et al. [31] found that ^18^F-FDG SUVmax in the bone marrow of patients with Hodgkin's lymphoma was significantly correlated with the hemoglobin level because of red marrow hyperplasia.

In our study, spleen SUV and liver SUV were directly compared with the hemoglobin level and both had a negative correlation with the hemoglobin level. However, our study showed that there was no significant correlation between the hemoglobin level and ^18^F-FDG uptake of marrow, which was not consistent with the results of the previous reports.

This study had several limitations because of its retrospective design. First, the sample size was relatively small. Further large-scale studies are needed to clarify the significance of diffuse ^18^F-FDG uptake in the spleen and liver. Second, diffuse splenic ^18^F-FDG uptake was not specific to the patient with anemia, as it was observed in many other diseases [32–35]. Third, none of the patients had follow-up PET/CT or blood routine examination studies. The correlations observed between the hemoglobin level and spleen SUV as well as liver SUV needed to be further assessed with long-term follow-up studies.

## 5. Conclusion

Our study revealed the correlation between diffuse splenic and hepatic ^18^F-FDG uptake and anemia in patients who underwent PET/CT. Imaging examination of anemia has important application value in a variety of clinical and research situations, and the results of our study may provide potential indicators and shed light on future research of the imaging diagnosis of anemia.

## Figures and Tables

**Table 1:** Clinical characteristics of the study cohorts.

Characteristics		Subjects (*n* = 44)
Age, years		58.31 ± 14.80

Sex, *n*	Man	19
	Women	25

Anemia grade, *n*	None	3
	Mild	14
	Moderate	23
	Severe	4

Disease, *n*	Colon cancer	7
	Esophageal cancer	4
	Cervical carcinoma	8
	Lung cancer	14
	Gastric cancer	5
	Pneumoconiosis	1
	Intracranial tumor	1
	None	7

**Table 2:** The correlations between the hemoglobin level and spleen SUV, liver SUV, marrow SUV, and the CTV of the LV cavity of the 44 cases.

Variable	Minimum	Maximum	Mean	SD	Hemoglobin	
					*r*	*P* value
Spleen SUVmax	1.295	3.675	2.01	0.55	*r*1 = −0.385	*p*1 = 0.01
Spleen SUVmean	1.129	3.458	1.743	0.489	*r*2 = −0.395	*p*2 = 0.008
Marrow SUVmax	0.815	4.841	2.146	0.731	*r*3 = −0.069	*p*3 = 0.656
Marrow SUVmean	0.676	3.36	1.761	0.539	*r*4 = −0.136	*p*4 = 0.377
Liver SUVmax	1.415	2.744	2.046	0.377	*r*5 = −0.365	*p*5 = 0.015
Liver SUVmean	1.126	2.387	1.658	0.33	*r*6 = −0.315	*p*6 = 0.037
CTV max	37	72	49.614	5.86	*r*7 = 0.33	*p*7 = 0.029
CTV mean	13	41	32.66	5.198	*r*8 = 0.382	*p*8 = 0.011
Hemoglobin (g/l)	54	169	85.82	25.188	NA	NA

## Data Availability

The data used to support the findings of this study are included within the article.
